# Utilisation of Pangolin (*Manis sps*) in traditional Yorubic medicine in Ijebu province, Ogun State, Nigeria

**DOI:** 10.1186/1746-4269-5-39

**Published:** 2009-12-04

**Authors:** Durojaye A Soewu, Ibukun A Ayodele

**Affiliations:** 1 Department of Plant Science and Applied Zoology, Olabisi Onabanjo University, PMB 2002, Ago-Iwoye, 120005, Ogun State, Nigeria; 2 Department of Wildlife and Fisheries Management, University of Ibadan, Ibadan, Oyo State, Nigeria

## Abstract

**Background:**

Concern about the use of endangered and threatened species in traditional medicine escalated as populations of many species plummeted because of poaching for the medicinal trade. Nigeria is known for a long and valued tradition of using wild animals and plants for medicinal purposes. Despite this, studies on medicinal animals are still scarce when compared to those focusing on medicinal plants. Utilisation of wild animals in traditional Yorubic medical practices was indiscriminate as it involved threatened species. By touting the medicinal properties of these species, traditional medicine fuel continuing demand, thereby subjecting such species to further threats. This paper examined the use and commercialisation of pangolins for traditional medicinal purposes amongst the Ijebus, South-western Nigeria, and the implications of this utilisation for the conservation of this species.

**Methods:**

Traditional Yorubic medical practitioners (tymps) (16) and dealers in traditional medicinal ingredients (56) in public markets in Ijebu province, Nigeria, were interviewed using open-ended questionnaires. The dynamic stock movement of pangolins in the stalls of dealers was also monitored to determine quantity of pangolins sold into the traditional Yorubic medicinal practices. Specific conditions treated and the parts required were also documented.

**Results:**

A total of 178 whole pangolin carcasses were sold into traditional medical practices. Above 55% of respondents had just primary education, over 90% of respondents were not aware of either the conservation status of this species or the existence of any legal machinery regulating its trade and utilisation, while 14% admitted to giving contracts to hunters for deliberate search for this animal when needed. More than 98% of respondents have no other means of livelihood. The trade was female dominated while the healing practice had more males. Pangolins were used in various preparations to treat a total of 42 conditions. These include infertility, gastro-intestinal disorders, safe parturition, stomach ulcers, rheumatism and fibroid. Traditional Yorubic medicine also accommodated some situations that are out of the range of conventional medicine like boosting sales, conferring invisibility, removing bad luck, appeasing/wading off witches cum evil forces and money rituals. Some of these situations specifically require juvenile, or even pregnant female animals.

**Conclusion:**

Traditional Yorubic medical practices eats deep into the reproductive base of the species, presently listed in Appendix II of CITES and Schedule I of the Nigerian Decree 11 (1985), both of which recommended strict control in sales and utilisation of this species. Its numerous medicinal values, folk culture and financial benefits of these activities are the main factors promoting the commercialisation and use of this species. Pharmacological studies on the various preparations are required to identify the bioactive compounds in them. There is a need for improved and urgent measures to conserve populations of this species *in-situ*. Massive education and enlightenment is urgently needed for the populace to have the necessary awareness and orientation about the conservation of this species.

## Introduction

Traditional medical system plays a key role in health care around the world [1]. Plants and animals have been used as medicinal sources since ancient times, and even today, animal and plant based pharmacopoeias continue to play an essential role in world health care [2]. The use of biological resources for various therapies has been documented in many different parts of the world, especially in remote regions where traditional medicines provide a *de facto *alternative to "modern" health care system [2]. The World Health Organisation estimated that 80% of the world population relies on traditional medicine prepared mainly by the use of natural products (animals and plants) to meet their daily health requirements [1,3-6]. Africa is known for a long and valued tradition of using wild animals and plants for medicinal purposes [7]. Traditional healing existed in Africa long before the advent of modern medicine and the people depended largely on traditional medicine as their only source of health care [8]. Traditional medicine as practiced today even made new drug discoveries, which have been found useful in curing major ailments that were previously incurable with orthodox medicine as well as other hereditary ailments such as diabetes mellitus and hypertension [9]. While stressing the importance of zootherapy to human kind the world over, [10] submitted that the time has come to record indigenous knowledge related to therapeutic animal uses and to devise strategies to exploit these natural resources more sustainably. People patronise traditional medicinal practices in all its ramifications due to lack of, or inadequacy of provisions of orthodox medical care. Traditional medical care is also more readily available to the majority of human population [10,11]. According to [4], traditional medicine is not viewed only as the best method for some treatments, the number of traditional medical practitioners (TMPs) practicing in most African regions is far greater than the number of orthodox medical practitioners, indicating that the availability of traditional medicine outweighs that of orthodox medicine. The 'engine' for traditional medicinal practices is the wild resources (flora and fauna) used extensively in preparation of 'herbal drugs' employed in the treatment of diseases and situations [12]. Although plants and plant- derived materials make up the majority of ingredients used in most traditional medical systems, whole animals, animal parts and animal derived products also constitute important elements of the folk pharmacopoeia throughout the world [2]. The World Health Organisation (WHO) stated that traditional medicine refers to health practices, approaches, knowledge and beliefs incorporating animal and mineral based medicines, spiritual therapies, manual techniques and exercises, applied singularly or in combination to treat, diagnose and prevent illnesses or maintain well-being [13]. Traditional medicine was further defined by WHO as the sum total of all knowledge and practices, whether explicable or not, used in diagnosis, prevention and elimination of physical, mental or social imbalance and relying exclusively on practical experiences and observations handed from generation to generation, whether verbally or in writing [14].

A common dilemma facing all fauna species is the soaring demand for their body parts for us in medicinal practices [15]. The continued depletion of medicinal wildlife resources not only embodies a challenge for conservation, but more importantly represents a serious threat to the health status of human population [4,12]. There is a risk that a growing herbal market and its great commercial benefit might pose a threat to biodiversity through the over-harvesting of the raw materials for herbal medicines and other natural health care products. These practices, if not controlled can lead to the extinction of endangered species and the destruction of natural habitats and resources [13]. Presently, the world is facing potentially massive loss of wildlife due to over-hunting and over-fishing. Regrettably, the demand created by traditional medicine is one of the causes of the over-exploitation of wild population of numerous animal species [16]

Yorubic medicine i.e traditional medical system based on the culture and mythological beliefs of the Yoruba people is indigenous to, and widely practiced on the African continent. Yorubic medicine has its roots in the Ifa Corpus, a religious text revealed by the mystic prophet, Orunmila, over 4,000 years ago in the ancient city of Ile-Ife, now known as Yorubaland [17]. Within the last 400 years, this healing system has also been practiced in the day-to-day lives of individuals in the Caribbean, and South America, in large part, because of the traditions brought over by African slaves arriving in the Americas [12,17].

The therapeutic use of animals and animal parts to treat human ailment has been much less studied than plants, [10,18]. The recent arousal of interest in zoo-therapy stems from rapid decline in number of neo-tropical fauna species. In search of therapeutic uses of the fauna species, the ecosystem has been greatly degraded, resulting to the extinction of most species well before they could be studied scientifically [19,20]. Certain animals are already becoming rare due to indiscriminate killing and, a record of indigenous knowledge related to therapeutic animal use is urgently required as a first step towards devising strategies for sustainable exploitation of these natural resources [11].

Pangolins have been reported as being used in traditional medical systems of many peoples around the world [8,15,11,21-23]. Pangolins were also reported as being among the species most consistently used for traditional medicine in Africa [22,23] and were even thought to be natural reservoir of magic and charms [24].

As regards conservation status, pangolins are presently rated as *near threatened *on IUCN *Red Data Book *[12]. All three western African species of pangolins are protected in Nigeria under Schedule 1 of Decree No. 11 (1985): Control of International Trade in Endangered Wild Fauna and Flora; while all four African species are listed in Class B of the 1986 African Convention on Nature and Natural Resources. This study was designed to identify the medicinal values of pangolin according to traditional Yorubic pharmacopoeia and determine the quantity of pangolin sold into traditional Yorubic medicinal practices over a period of time, so as to have an insight into the utilisation pressure on wild populations of this animal.

## Materials and methods

**Figure 1:**
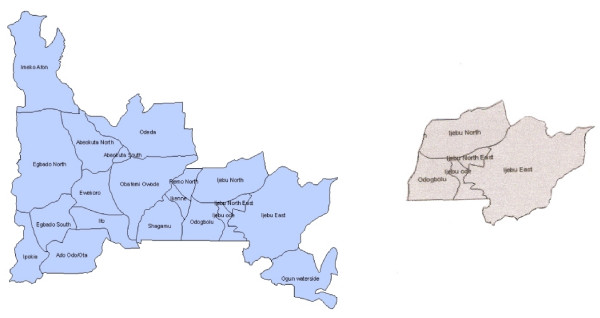
**The Map of Ogun State showing study site**.

The study was conducted in four local government areas of Ijebu province in Ogun State. Dealers in traditional medicine ingredients and traditional Yorubic medical practitioners (tymps) were randomly selected in four towns: Ago-Iwoye, Ijebu-Ode, Ijebu-Imushin, and Imodi-Imosan representing respectively Ijebu-North, Ijebu-East, Ijebu-Ode and Odogbolu local government areas as shown in figure 1.

Ogun State is entirely in the tropics. Located in the Southwest Zone of Nigeria with a total land area of 16,409.26 square kilometres, it is bounded on the West by the Benin Republic, on the South by Lagos State and the Atlantic Ocean, on the East by Ondo State, and on the North by Oyo and Osun States. It is situated between Latitude 6.2°N and 7.8°N and Longitude 3.0°E and 5.0°E. It has an estimated population of 3,486,683 people for the year 2005 [25].

A preliminary survey was conducted between December 2006 and January 2007 to standardise the questionnaire. During the main survey between April-July 2007, a total of 56 dealers in traditional medicine ingredients at markets in selected towns were interviewed using open-ended questionnaires. Ago-Iwoye (10), Ijebu-Ode (26), Ijebu-Imushin (15), and Imodi-Imosan (5). The dynamic flow of sales in pangolins was recorded using the head count method [12]. In addition, 16 randomly selected traditional Yorubic medical practitioners (ymps) were interviewed in the selected towns.

## Taxonomy

Scientific classification

Kingdom:         Animalia

Phylum:           Chordata

Class:              Mammalia

Infraclass:       Eutheria

Superorder:     Laurasiatheria

Order:             Pholidota

Family:            Manidae

Genus:            Manis

Species

Manis culionensis

Manis gigantea

Manis temminckii

Manis tricuspis

Manis tetradactyla

Manis crassicaudata

Manis pentadactyla

Manis javanica

### Local name in Yoruba: aika, arika or akika

Pangolin, or scaly anteater, is the common name for African and Asian armoured mammals comprising the order Pholidota, characterized by a long and narrow snout, no teeth, a long tongue used to capture ants and termites, short and powerful limbs, a long tail, and a unique covering of large, overlapping body scales. There is only one extant family (Manidae) and one genus (Manis) of pangolins, comprising eight species. There are also a number of extinct taxa [26].

Found in diverse habitats in tropical and subtropical regions in sub-Saharan Africa and southern and south-eastern Asia, pangolins are important parts of terrestrial food chains, consuming insects (largely ants and termites) and being consumed by leopards, lions, tigers, hyenas, and pythons. Their unique form and behaviours, including rolling up into a pine-cone like ball when threatened, add greatly to the wonder of nature. In addition, they are hunted and trapped for their meat, skin, and scales.

The name "pangolin" derives from the Malay word pengguling ("something that rolls up"). The order name, Pholidota, means "the scaled animal" [26]

## Description

Pangolins are similar in appearance to anteaters in that they have a long and tapered body shape and snout, a very long, worm-like tongue, short and powerful limbs, and no teeth. The size of pangolins varies by species, with a head and body length ranging from 30 to 90 centimetres (12 to 35 inches), a tail of from 26 to 88 centimetres (10 to 35 inches), and a weight from about 1 to 35 kilograms (2 to 77 pounds). Females are generally smaller than males. The males may weigh ten to fifty percent more.

The physical appearance of pangolins is marked by large, hardened, overlapping, plate-like scales covering their skin, making them almost reptilian-looking. The scales, which are soft on newborn pangolins but harden as the animal matures, are made of keratin, the same material of which human fingernails and tetrapod claws are made. Pangolins are distinct among mammals in terms of this unique adaptation of a covering of keratin body scales [27]. The pangolin is often compared to a walking pine cone or globe artichoke. It can curl up into a ball when threatened, with its overlapping scales acting as armour and its face tucked under its tail. The scales are sharp-edged, providing extra defence [26,27].

The scale colour, size, pattern, quantity, and shape vary among the different species and also can differ somewhat among individuals within a species. Generally, there are 18 rows of overlapping scales around the body, with scales continuous to the tip of the tail. The African species differ from the Asian by having a double row starting two-thirds of the way to the tip of the tail. Coloration can vary from dark brown to yellowish, and include dark olive-brown, pale live and yellow brown. The number of scales remains constant throughout life [27]. Scales from one adult animal weigh an average of 1 kg. [28,29].

Parts of the body without scales (underside of head, sides of face, throat, and neck, stomach, inner sides of limbs, and snout and chin) are thinly covered with hair. The Asian species have three or four hairs at the base of each scale, but the African species lack hairs at the base of the scales [26]

The limbs of pangolins are short but powerful and are tipped with sharp, clawed digits: the middle digit is the largest. The front claws are large and useful for digging into termite mounds and ant hills. However, the front claws are so long that they are unsuited for walking, and so the animal walks with its fore paws curled over to protect them.

The heads of pangolins are small and tapered, and the eyes are small. Depending on the species, the ears may be rudimentary or absent. They have poor vision and only average hearing. The jaw lacks teeth, although embryos have small, temporary, primordial teeth. They do have an excellence sense of smell [27].

The tongues of pangolins are extremely elongated, may be round or flattened, and extend into the abdominal cavity. The tongue is unattached from the hyoid bone and extends past the pharynx deep into the thorax, as with the giant anteater and the tube-lipped nectar bat. This extension lies between the sternum and the trachea. Large pangolins can extend their tongues as much as 40 centimetres (16 inches), with a thickness of only 0.5 centimetres (1/4 inch). The very large salivary glands coat the tongue with a sticky saliva for capturing insects[26]

The tail is powerful and mobile, and is fully prehensile in arboreal species, despite being covered with scales. The tails of terrestrial species is shorter and more blunt and is not considered fully prehensile [27].

For defensive purposes (in addition to rolling into a ball), pangolins can emit a noxious smelling musky fluid from glands near the anus, similar to the spray of a skunk.

## Distribution and habitat

Pangolins are found in tropical and subtropical regions of Africa and Asia. They are found south of the Sahara in Africa and in southern and south-eastern Asia, including India, Thailand, Myanmar, southern China, the Malay Peninsula, Indonesia, the Philippines, and various other islands [27].

Pangolins inhabit diverse habitats, including rainforest, deciduous forest, grassland, steppes, open country, thick bush, and shrubby slopes, as long as they contain ants and termites [26-29].

## Behaviour, diet, and reproduction

Pangolins include both terrestrial (ground-dwelling) and arboreal (tree-climbing) species. Some arboreal pangolins live in hollow trees, whereas the ground dwelling species dig tunnels underground, up to a depth of 3.5 meters (11 feet). Some species can dwell on both the ground and in trees, although they are classified as either terrestrial or arboreal. Most are good climbers and pangolins are also good swimmers [27]

Pangolins are nocturnal animals, using their well-developed sense of smell to find insects. The long-tailed pangolin (*Manis tetradactyla*) is also active by day. Pangolins spend most of the daytime sleeping, curled up into a ball.

Pangolins lack teeth and the ability to chew. Instead, they tear open anthills or termite mounds with their powerful front claws and probe deep into them with their very long tongues. Some species, such as the tree pangolin, use their strong tails to hang from tree branches and strip away bark from the trunk, exposing insect nests inside [26,27].

Pangolins tend to be shy, solitary, and unsociable creatures, and slow and deliberate movers. However, all species can move quickly. When confronted, they will roll up into a ball with the sharp-edged scales offering protection, and movements of the tail and scales deter predators. Both urine and the posterior gland secretions are expelled as deterrents as well. [27]

Insectivorous animals, pangolins have a diet almost exclusively one of insects, mostly ants and termites, but also some soft-bodied insects and larvae. Their strong sense of smell is employed in finding their prey. Some species have a strong preference for particular species of ants or termites [26].

Gestation is 120-150 days. African pangolin females usually give birth to a single offspring at a time, but the Asiatic species can give birth to between one and three offsprigs at a time. Weight at birth is 80 to 450 grams (3-18 ounces), and the scales are initially soft. The young cling to the mother's tail as she moves about, although, in burrowing species, they remain in the burrow for the first 2 to 4 weeks of life. Weaning takes place at around three months of age, and pangolins becomes sexually matured at two years. In *Manis tricuspis*, the young stayed with their mother for about five months [26,28,29].

With majority of its uses attributed to its scales and meat, other uses of pangolins include use in food as a supplementary protein source and as adornments [12,23].

## Carcass Quantification

To determine the number of carcass of the species that passed through the stalls for the period, the number of carcasses sold out between consecutive market days were taken and summed up.

The main attributes of market dynamics measured during this survey include the quantity of pangolin utilised by Yorubic medical practices as revealed by sales figures for the species i.e. carcass number and the average sales figure per stall/dealers for this species.

The whole animal or the animal head seen at each stall on the first visit were counted and recorded and this was taken as the initial opening stock [12]. During subsequent visits to the stall, the remnant numbers were counted and recorded. Also the number supplied to the stall after the last count was noted for the species. This allows for the determination of the actual number sold out during the period using:

number sold out = opening balance + added stock - closing balance.

## Medicinal Values of Pangolins

Traditional Yorubic medical practitioners provided information on the medicinal values of the species i.e. the specific conditions in which preparations made from pangolins parts are used, the actual part required and other preferences especially age and gender required in the various preparations.

## Results and Discussion

The trade was female dominated (95%), while the healing practice had more males (94%). More than 73% of respondents were aged between 40 and 70 years while 13% were above 70 years in age (table 1). Above 9 percent of respondents had no formal education, 4 percent had exclusive quoranic education, 56 percent had only primary education while less than 6 percent had post secondary education as shown in table 2. Few respondents however combined quoranic with western education. Table 3 showed the source of pangolins to the dealers and tymps. Most pangolins sold in the various markets were procured either from wholesale markets located outside the state or from intermediaries/middlemen sellers as only 1.39 percent of the respondents were involved in direct cropping from the wild. All respondents submitted that the primary source of this animal can only be from populations in the wild as there are no records of any ex-situ conservation project for the species in this part of the world. This submission agreed with the findings of [4] which recorded that all the species of plant and animals traded for traditional medicinal practices in South-Africa came from the wild. More than sixty-two percent of respondents engaged in prepayment for pangolins, above twenty-three percent procured the animals by chance/on encounter while about fourteen percent admitted to contracting hunters or poachers for deliberate searches for the animals as shown in table 4. Contract hunting is employed when there is an urgent need for the animal, whole or parts, but which is not readily available in nearby markets. Higher incentives often attached to contract hunting encourage poachers to push deeper into the natural habitat for this animal. In addition, this practice may inadvertently promote destruction of the habitat during desperate searches, thereby exposing individuals remaining in the population to further risks. More than ninety-five percent of respondents had no awareness at all about conservation status of pangolin, the restriction placed on its trade as well as the various legal structures conferring protection on this animal (table 5). This very low level of awareness cannot be totally divorced from the generally low level of literacy in the country. Also, above ninety-percent of respondents were of the opinion that wild animals are nature's gift that must be utilised as long as it is available. Hence, they expressed apathy towards supporting the conservation of this animal through sustainable utilisation as they feel this may threaten their only means of livelihood (table 6). Such an attitude poses a great danger for sustainability as far as this species is concerned. Table 7 gives some of the identified conditions for which pangolin-based preparations are used to treat and the parts and/or other attributes of the animal required for desired efficacy.

**Table 1:** Gender and age distribution of respondents

Gender	Dealers	%	YMPs	%	All respondents	%
Male	3	5.4	15	93.8	18	25
Female	53	94.6	1	6.2	54	75
**Total**	56	100	16	100	72	100
**Age**						
18-25	04	7.1	-	-	04	5.6
25-40	13	23.2	2	12.5	15	20.8
40-55	21	37.5	3	18.8	24	33.3
55-70	11	19.6	9	56.3	20	27.8
>70	07	12.5	2	12.5	09	12.5
**Total**	56	100	16	100	72	100

**Table 2:** Level of Education of the Respondents

Level	Dealers	%	YMPs	%	All respondents	%
None	4	7.1	3	18.8	7	9.7
Quoranic	1	1.8	2	12.5	3	4.2
Primary	33	58.9	7	43.8	40	55.6
Secondary	15	26.8	3	18.8	18	25.00
Post primary	3	5.4	1	6.3	4	5.6
**Total**	56	100	16	100	72	100

**Table 3:** Source of Animal

Source	Dealers	%	YMPs	%	All respondents %
Direct cropping	0	0	1	6.3	1.4
Wholesale markets	43	76.8	3	18.8	63.9
Retailers	13	23.2	12	75	34.7
Total	56	100	16	100	100

**Table 4:** Mode of Procurement

Mode	Dealers	%	YMPs	%	All respondents %
By chance	17	30.4	0	0	23.6
Prepayment	32	57.1	13	81.3	62.5
Contract deliberate hunting	7	12.5	3	18.8	13.9
Total	56	100	16	100	100

**Table 5:** Awareness of Conservation status of Pangolins

Awareness	Dealers	%	YMPs	%	All respondents %
**None**	54	96.4	15	93.8	95.8
**Low**	2	3.6	1	6.25	4.2
**Moderate**	0	0	0	0	0
**High**	0	0	0	0	0

**Table 6:** Willingness to support Conservation of Pangolins

Support level	Dealers	%	YMPs	%	All respondents %
None	52	92.9	13	81.3	90.3
Low	2	3.6	1	6.3	4.2
Moderate	1	1.8	1	6.3	2.8
High	1	1.8	1	6.3	2.8

**Table 7:** Parts of animal used, age and gender preference and, conditions treated with Pangolins

S/N	Parts required	Age preference	Gender preference	Conditions treated
1	Scales	-	-	To cure stomach disorders (Ogun Inu rirun, arunsu)
2	Head	Adult	-	To remove dizziness (Ogun oyi oju ati inu)
3	Scales	-	-	To cure stomach ulcers (Ogun ogbe inu)
4	Head	-	-	To arrest thieves or protect properties (Imole, Arimole, Kikanse Ole)
5	Scales	-	-	To cure gonorrhoea (Ogun Atosi)
6	Whole animal	Adult	Pregnant Female	To remove barreness in woman (Ogun idaduro fun obinrin laganlagan)
7	Whole animal	Newborn	-	For invisibility (isiju iriran ariran)
8	Whole animal	Adult	Female	To enable womb to retain semen (ogun eda obirin)
9	Whole animal	-	-	For good sales of markets (wares) (awure itaja)
10	Whole animal	Juvenile	Female	To hypnotise female for sexual abuse (Amudo)
11	Whole animal	Adult	Pregnant Female	To prevent miscarriage (ogun oyun dide yiya)
12	Whole animal	Adult	Pregnant Female	To remove fibroid tissue (ogun oyun iju)
13	Whole animal	Adult	Female	To regulate menstrual period (ogun alase) and aid pregnancy (ogun aremo)
14	Whole animal	Adult	Pregnant Female	For foetus movement and development (ogun oyun jiji, didagba)
15	Whole animal	Adult	Female	Love potion (Iferan), to win, seduce, commandeer woman for marriage (fife, gigba obirin)
16	Whole animal	Adult	Female	For good delivery (Ebi, ogun awebi, bibi alaboyun)
17	Whole animal	Adult	Pregnant Female	To eject (deliver) overdue pregnancy (ogun oyun pipe)
18	Whole animal	-	-	Against sexual acts in dreams (ogun esunkun, oko orun)
19	Whole animal	Adult	Male	To aid male potency (ogun aleko, afato)
20	Whole animal	-	Female	To control womb (waist) worms in women (ogun okinisa-latan latan)
21	Scales	-	-	To regulate menstrual period (ogun alase)
22	Whole animal	Adult	-	Money rituals (asiri owo)
23	Scales	-	-	To cure male/female genital itching/swelling problem (ogun eyo okunrin/irobo obirin)
24	Scales	-	-	To cure or relief female vaginal swelling (ogun irobo tabi ido obinrin)
25	Scales	-	-	Healing of wounds/cuts
26	Flesh	Adult	-	Calmness of hardened mind.(Eyo nu).
27	Flesh/Scales	-	-	Protection
28	Scales	Adult	Female	Wading off witches/evil forces
29	Eyes	Juvenile	-	Kleptomania
30	Scales/Flesh	-	-	Mental illness
31	Scales/Flesh	-	-	Antidote/cure for sexual poison (magun)
32	Head	Juvenile	Female	Wading off/curing bad luck
33	Scales/Bones	-	-	Stroke
34	Scales	-	-	Anti-poison
35	Head	-	-	Convulsion
36	Scales	Adult	Female	Preventing witchcraft attack
37	Bones	Adult	-	Back pain
38	Scales	-	-	Aphrodisiacs/potency for men
39	Bones	-	-	Rheumatism
40	Scales	-	-	Amulets
41	Whole animal	Adult	Female	Appeasing witches and evil spirits
42	Whole animal	Adult	-	Prevention of curses, spells and hypnosis

**Figure 2:**
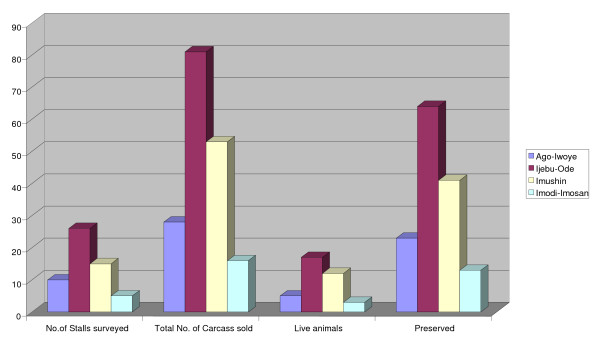
**Stalls and sales distribution**.

**Figure 3:**
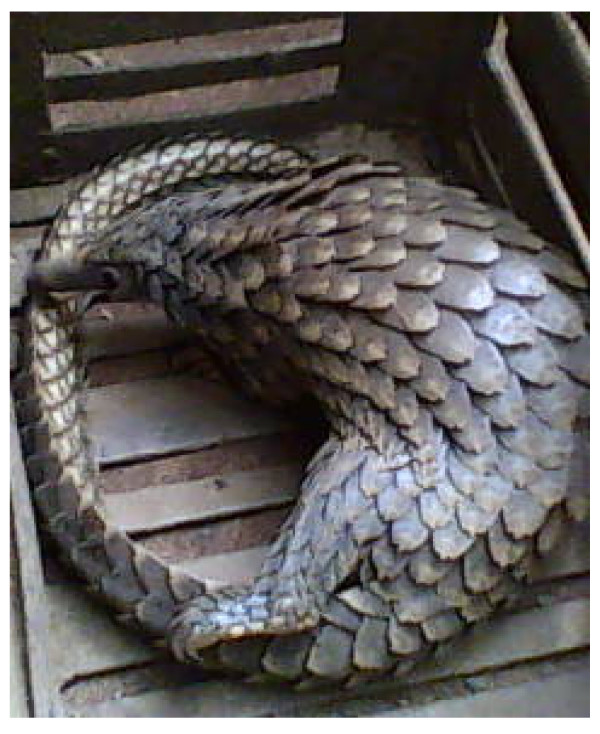
**Live pangolin on display**.

*Manis tricuspis *was the only species encountered during the market survey. One hundred and seventy-eight whole pangolins were sold out to traditional Yorubic medical practices during the period of this study in the order presented in figure 2, thirty-seven of which were live animals, one of which is shown in figure 3.

A pregnant female animal was required to treat some situations including barrenness in a woman, prevention of miscarriage, to remove fibroid tissue in women, ante-natal care, and ejection of prolonged pregnancy. Whole juvenile animals were used to prepare charms that can confer invisibility and hypnotise females for sexual abuse; eyes of a juvenile animal is utilised to treat kleptomania while the whole head of a juvenile pangolin was used to treat bad luck traits. Scales of pangolin were used to treat stomach disorders including ulcers, venereal diseases and genital swellings, and to regulate menstrual period in a woman. Pangolin scales were also used to prepare aphrodisiacs/potency medicine in men, treat stroke, mental disorders and external wounds. Scales of this animal were also used for other situations like preparing antidote for food poisons, sexual poisons "magun" meaning "do not climb", prepare charms that confer protection from spiritual attacks and wade off evil spirits and witches. Sexual poisons were designed to discourage infidelity in married women as any man who has intercourse with a laced woman suffers physiological disorders that may lead to death if not promptly and adequately treated. Whole female adult, not necessarily pregnant is employed in situations like enabling womb to retain semen, love potions to win a woman's heart, aid safe delivery and preparations to enhance fertility in a woman. Some of the other situations treated do not have specific age or gender preferences.

Diversity of uses of pangolins in Yorubic medicine agreed with the findings of several previous authors as reported for many peoples of the world. Some group of people in East India utilised the scales to make rings for personal use as charms or cures for haemarroids, rheumatism or labour pain. Some other groups in East India utilised the fat and brain for same conditions [3]. According to the Korean pharmacopoeia, pangolin scales are believed to help menstruation and breast milk circulation [3]. The Chinese believe a scale of pangolin reduces/subdue swellings and promote the drainage of pus, promote blood circulation and help breast-feeding women to produce milk. When mixed with the bark of certain trees, the scales are thought to neutralise witchcraft and evil spirits. If buried near a man's door, they are said to give an interested woman power over him [24,12]. Sometimes the scales are burnt to keep lions and other wild animals away [30]. Tibet in their traditional Tibetan incense makes use of pangolin scales [31]. During the Tibet purification "Ribo Sangcheo" pangolin scales called "Nagi" is utilised. Also the Tibet energising "Lungta" utilised the scales of pangolin [31].

The Chinese has a wide range of other uses of the scale of pangolin in Traditional Chinese Medicine. In curing Amenorrhea, in removing masses in the abdomen, to cure Arthralagia, as Galactostatis, to cure sore, carbuncle and other pyogenic skin infections [32].

Pangolin scales had also been reported to have cured various skin diseases [12]. The scales and blood are used to treat a variety of conditions such as heart problems; good luck charms; for rain making; to protect against bad omens and for 'bullet-proofs' charms [4].

The average sale figure of 1.06 carcasses per dealer in a month is obviously beyond sustainability for a species listed in appendix II of CITES listing and schedule 1 of the Nigeria's Endangered Species (Control of International Trade and Traffic) Decree No 11, 1985. These listings imply that though not necessarily threatened with extinction, trade in this species must be controlled in order to avoid utilisation incompatible with their survival. The open unregulated sales of pangolins in markets during this study show either a total lack of awareness of the existence and implication of *Decree No 11*, or that the respondents (dealers and tymps) know that the law is not enforced. With no successful record of captive breeding or domestication yet in this part of the world, the only source of this animal is from populations in the wild that are already fast declining due to over-exploitation for medicinal trade. At prices ranging from 1,500-2000NGN per carcass, the trade as recorded in this study was worth 267,000-356,000NGN. For a trade that involves near-zero production cost outside that of cropping the animal from the wild, there are enough incentives to hunt this animal (table 4).

With regards to the parts of the animal utilised, the scales recorded the highest fidelity level i.e frequency of occurrence in uses, occurring in virtually all categories of the preparations. Another factor that calls for concern is that utilisation of pangolin in Yorubic medicine readily cuts across all age grades or classes of the animal: juvenile, sub-adults and adults. Some situations specifically demand for and utilised juvenile animals. For instance, the preparation of charms that will make someone invisible at will often require juvenile animals that have not been exposed to sunlight. Other conditions require the use of specific gender, especially preparations to treat infertility in women will demand for a matured, sometimes even pregnant female animal. This practice eats deep into the basal pool for this species as it substantially reduces the number of individuals that will have the chance to participate in reproductive activities.

## Conclusion

Utilisation of pangolin in traditional Yorubic medicine is very diverse as far as situations treated are concerned. There is a need to investigate the population size of this species in the wild in Nigeria as respondents are already complaining of increasing difficulty in getting the animal from the various sources. Also there is a need to examine the properties that confer so much curative potencies on the scales. This requires pharmacognosy investigations to determine the bioactive compounds in the scales. The desired increase in the awareness of people regarding conservation of natural resources requires mass enlightenment and education of the populace. The essence and implications of Decree 11 (1985) should also be accorded the publicity it deserves if it is expected to make any meaningful contribution to conservation of natural resources. Communities that are hosts to conservation programmes especially in-situ projects should be actively involved in the design and management of such schemes as a sense of joint ownership with the appropriate authorities would encourage them to fully co-operate and participate in such projects. Capacity building in diverse forms for member of these communities will also be more effective in enjoining their supports to ensure success of conservation efforts.

## Competing interests

The authors declare that they have no competing interests.

## Authors' contributions

DAS conceived of the study, participated in its design and coordination, conducted the preliminary and main survey and drafted the manuscript. IAA participated in the design of the study, review of literature and revision of the manuscript. Both authors read and approved the final manuscript.

## References

[B1] Lee SK Trade in traditional medicine using endangeredspecies: An International Context. Paper presented at "Healthy people -healthy wildlife; the secondAustralian symposium on traditionalmedicine and wildlife conservation Traffic Report 1999 http://www.traffic.org

[B2] Alves RRN Lima HN Tavares MC Souto WMS Barbozaand Vasconcellos A Animal-based remedies as complimentary medicines in Santa Cruz do Capibaribe, Brazil BMC Complimentary and Alternative Medicine 2008 8 44 10.1186/1472-6882-8-44 PMC2503950 18647413

[B3] Kang S Phipps M A Question of Attitude: South Korea's Traditional Medicine Practitioners and Wildlife Conservation A TRAFFIC East Asia Report 2003

[B4] Marshall NT Searching for a cure: conservation of medicinal wildlife resources in East and southern Africa TRAFFIC International 1998

[B5] Akerele O Summary of WHO guidelines for the assessment of herbal medicines Herbal Gram 1993 28 13 20

[B6] Bodasing A Overview of wildlife trade issues in sub-saharan Africa TRAFFIC Report 1999 http://www.traffic.org

[B7] Anon: Time to act on traditional medicine and wild resources: A challenge to the health and wildlife heritage of Africans TRAFFIC Reports 1999 http://www.traffic.org

[B8] Adeola MO Importance of wild animals and their parts in the culture, religious festivals and traditional medicine in Nigeria Environmental Conservation 1992 19 25 134 10.1017/S0376892900030605

[B9] Odu MO The art of traditional healing in Nigeria National Concord 1987 5

[B10] Costa-Neto E Traditional use and sale of animals as medicines in Feira de Santana city, Bahia, Brazil Indigenous knowledge and development monitor articles 1999 7-2 1 4

[B11] Kakati LN Doulo V Indigenous Knowledge System of Nagal and India Journal of Human Ecology 2002 13 6 419 423

[B12] Soewu DA Utilisation of Wild Animals in Traditional Medicine in Ogun State, Nigeria PhD thesis 2006 University of Ibadan, Department of Wildlife and Fisheries Management

[B13] World Health Organisation Traditional Medicine 2003 http://www.worldhealthorganisation.com

[B14] Adesina SK Traditional Medical Care in Nigeria 2007 http://www.onlinenigeria.com/links/LinksReadPrint.asp?blurb=57417646686

[B15] Soewu DA Wild Animals in ethnozoological practices among the Yorubas of soutwestern Nigeria and the implications for biodiversity conservation African Journal of Agricultural Research Vol 2008 3 6 421 427

[B16] Alves RRN Rosa IL Why study the use of animal products in traditional medicines Journal of Ethnobiology and Ethnomedicine 2005 1 5 10.1186/1746-4269-1-5 16270931 PMC1277085

[B17] Sawandi T Yorubic Medicine: The Art of divine Herbology 2006 http://www.planetherbs.com

[B18] Alves RR Fauna used in popular medicine in Northeast Brazil Journal of Ethnobiology and Ethnomedicine 2009 5 1 10.1186/1746-4269-5-1 19128461 PMC2628872

[B19] Huxtable RJ The pharmacology of extinction Journal of Ethnopharmacology 1992 37 1 11 10.1016/0378-8741(92)90002-9 1453701

[B20] Gaski LA Johnson KA Prescription for extinction: Endangered Species and Patented Oriental Medicines in Trade Traffic USA 1994 300

[B21] El-Kamal HH Folk medicinal use of some animal products in Central Sudan Journal of Ethnopharmacology 2000 72 279 282 10.1016/S0378-8741(00)00209-9 10967482

[B22] Sodeinde OA Soewu DA Pilot Study of the Traditional Medicine Trade in Nigeria TRAFFIC Bulletin 1999 18 1 35 40

[B23] Brautigam A John H Tamsin H Jonathan H Recent Information on the Status and Utilisation of African Pangolins TRAFFIC Bullettin 1994 15 1 15 22

[B24] African Wildlife Fund.(AWF) Pangolin 2006 http://www.awf.org/content/wildlife/detail/1017

[B25] Anon Official website of Ogun State 2009 http://www.ogunstate.gov.ng/eGovernment/index.php?option=com_content&view=article&id=65&Itemid=67

[B26] New World Encyclopedia 2007 http://www.newworldencyclopedia.org

[B27] Atkins WA Grzimek B, Kleiman DG, Geist V, McDade MC Pholidota pangolins (Manidae) Grzimek's Animal Life Encyclopedia 2004 16 Detroit: Thomson-Gale ISBN 0787657921

[B28] Fahey B "Manis tricuspis" (On-line), Animal Diversity 1999 http://animaldiversity.ummz.umich.edu/site/accounts/information/Manis_tricuspis.html

[B29] Wikipedia, Pangolin 2006 http://en.wikipedia.org/wiki/Pangolin

[B30] Anon, Pangolin Trade in East India TRAFFIC Dispathches, No.4 2000 http://www.traffic.org/species-reports/traffic_species_mammals51.pdf

[B31] Kyle L Resources for Living Well Traditional Tibetan Incense 2005 http://www.essentialoil.com/incense.html

[B32] Lam J Chinese Traditional Medicine. MyHealthspan.com 2006 http://www.myhealthspan.com/PangolinScales.shtm

